# The importance of using the oral glucose tolerance test when assessing the glycemic profile in patients with adrenal incidentaloma

**DOI:** 10.3389/fcdhc.2025.1659353

**Published:** 2025-10-02

**Authors:** Julia Magarão Costa, João Felipe Dickson Rebelo, Andrea Faria Dutra Fragoso Perozo, Fernanda Damasceno Junqueira, Ana Beatriz Alcantara Bérenger Samarcos De Almeida, Juliana Saldanha Milaresco, Michelle dos Santos Souza, Aline Barbosa Moraes, Joana Rodrigues Dantas, Leonardo Vieira Neto

**Affiliations:** 1 Department of Internal Medicine and Endocrine Unit, Universidade Federal do Rio de Janeiro, School of Medicine, Hospital Universitário Clementino Fraga Filho, Rio de Janeiro, Brazil; 2 Department of Internal Medicine and Nutrology Unit, Universidade Federal do Rio de Janeiro, School of Medicine, Hospital Universitário Clementino Fraga Filho, Rio de Janeiro, Brazil; 3 School of Medicine, Universidade Federal do Rio de Janeiro, Rio de Janeiro, Brazil; 4 Department of Clinical Medicine and Endocrine Unit, Universidade Federal Fluminense, School of Medicine, Hospital Universitário Antônio Pedro, Niterói, Brazil

**Keywords:** adrenal incidentaloma, adrenal adenoma, diabetes mellitus, prediabetes, oral glucose tolerance test

## Abstract

**Introduction:**

There are limited data regarding the role of oral glucose tolerance test (OGTT) in classifying glycemic alterations in patients with adrenal incidentaloma (AI). This study aims to compare the frequency of dysglycemia [pre-diabetes mellitus (DM) and DM] among patients with non-functioning adrenal incidentalomas (NFAI), mild autonomous cortisol secretion (MACS), and controls; and to assess the area under the curve (AUC) in the OGTT and determine whether the OGTT was decisive in diagnosing dysglycemia in each population.

**Methods:**

A cross-sectional study was conducted on 65 NFAI (1mg-dexamethasone suppression test [DST] ≤1.8μg/dL), 45 MACS (1mg-DST ≥1.9μg/dL), and 56 controls. The control group was selected based on normal adrenal imaging. Patients were classified as normoglycemic or dysglycemic based on fasting glucose, glycated hemoglobin, and OGTT.

**Results:**

AUC >290mg.h/dL was found in 75% of MACS, 55% of NFAI, and 22% of controls (p**=**0.008). The presence of AI was determinant for this result. Glucose levels ≥155 mg/dL at the 1-hour during the OGTT were observed in 75% of MACS, 65% of NFAI, and 28% of controls (p=0.01). Dysglycemia frequency was higher in MACS and NFAI than controls (91.1 *vs*. 90.8 *vs*. 73.2%; p=0.01). The OGTT changed the classification in 27% of MACS, 23% of NFAI, and 3% of controls (p=0.03). Presence of AI increased the odds ratio for benefiting from OGTT to obtain a more accurate dysglycemia classification by 9.5 times.

**Conclusion:**

Patients with AI had a higher dysglycemia frequency, and a significant number of these patients benefited from OGTT in classifying glycemic alterations.

## Introduction

Adrenal incidentaloma (AI) is an adrenal mass incidentally discovered on imaging studies performed for reasons unrelated to adrenal diseases (1, 2). The incidence of AI has been increasing significantly with the advancement of imaging technology and its wider availability (3, 4). In most cases, these lesions are benign, non-functioning adenomas. The most common functioning adrenal lesion is mild autonomous cortisol secretion (MACS), previously known as subclinical Cushing’s syndrome or subclinical hypercortisolism (1, 3).

Recent AI guideline management suggests using the 1mg-dexamethasone suppression test (1mg-DST) to categorize patients as having non-functioning adrenal incidentaloma [NFAI, serum cortisol level ≤50 nmol/L (≤1.8 μg/dL)] or MACS [> 50 nmol/L (>1.8 μg/dL)] (1).

While MACS is recognized for its association with metabolic disturbances, more recent literature has also shown an increased prevalence of cardiometabolic comorbidities in patients with NFAI. Patients with MACS have a higher incidence of central obesity, impaired glucose tolerance (IGT), metabolic syndrome (MS), and diabetes mellitus (DM). Recent studies have also shown an increase in these conditions in NFAI (5–9).

Regarding glycemic alterations, current guidelines for the management of AI do not explicitly link NFAI to these comorbidities. While MACS is recognized as a condition associated with IGT and DM, there is currently no specific recommendation for screening these disorders in this population (1–4). In patients with overt hypercortisolism, the oral glucose tolerance test (OGTT) with 75g of dextrose is considered of paramount importance to diagnose glycemic disorders. This exam becomes particularly important in this population since fasting glucose (FG) levels may not be sufficient to assess glucose homeostasis in Cushing’s syndrome, since most of these patients have normal FG levels (10). It is extremely important to highlight the role of this test in these patients, since the OGTT is an exam that is often neglected in clinical practice, especially when FG is normal (10). However, studies demonstrating the importance of OGTT in patients with AI are still lacking (10, 11).

This study aims to investigate the frequency of prediabetes and DM in patients with MACS and NFAI, compared to a control group. Additionally, the study seeks to determine whether OGTT can be a decisive tool for accurately classifying glycemic alterations in these patients.

## Methods

### Study design

This is a cross-sectional observational study carried out at Clementino Fraga Filho University Hospital (HUCFF) of the Federal University of Rio de Janeiro (UFRJ). The study was approved by the local Research Ethics Committee under protocol number 37024120.6.0000.5257 and was conducted in accordance with the Helsinki Declaration (2013). All subjects provided written informed consent before participating in the study, including consent for the publication of the results.

From February 2020 to August 2022, patients were selected from the Endocrinology Outpatient Clinic at HUCFF/UFRJ, and the control group was selected using the hospital imaging database obtained from the Radiology Unit (patients with normal adrenal glands on abdominal computed tomography (CT) or magnetic resonance imaging (MRI).

The inclusion criteria for patients with AI were: 1) unilateral or bilateral adrenal mass detected incidentally during imaging examinations unrelated to suspected adrenal disease; 2) tumor with benign radiological characteristics, including a homogeneous mass with a regular shape and well-defined margins, and an attenuation index < 10 Hounsfield units. For tumors with a diameter ≥ 4 cm, contrast-enhanced washout CT was evaluated, and patients were included if the relative washout was >40%; 3) absence of signs and symptoms suggestive of overt Cushing’s syndrome, such as violaceous striae, delayed wound healing, and proximal muscle weakness; 4) absence of other diagnoses of adrenal tumors such as primary aldosteronism (PA), pheochromocytoma, paraganglioma, adrenocortical carcinoma, myelolipoma, or congenital adrenal hyperplasia; and 5) absence of medication use that interferes with dexamethasone metabolism such as barbiturates, phenytoin, carbamazepine, rifampin, pioglitazone, ketoconazole, ritonavir, macrolide antibiotics, estrogens, diltiazem and fluoxetine.

Patients and controls were evaluated according to the following exclusion criteria: 1) age <18 years; 2) pregnancy; 3) alcoholism or drug abuse; 4) hepatic or renal failure (defined as Child-Pugh score B or C and creatinine clearance <15mL/min, respectively); 5) uncontrolled neuropsychiatric disorders; 6) septic or seriously ill patients; 7) post-transplant; 8) current malignancy; and 9) glucocorticoid use.

The work up for AI was performed in all patients according to the 2023 European Society of Endocrinology guideline (1). The investigation of pheochromocytoma and PA was performed in accordance with the current guidelines (12, 13). The evaluation of MACS was performed using the 1mg-DST in all patients with AI as follows: patients with cortisol levels following 1mg-DST ≤50 nmol/L (≤1.8 μg/dL) after an overnight fast and collected between 07:00 and 09:00 AM were considered to have NFAI, whereas patients with cortisol levels >50nmol/L (>1.8 μg/dL) were considered to have MACS (1).

**Figure 1:**
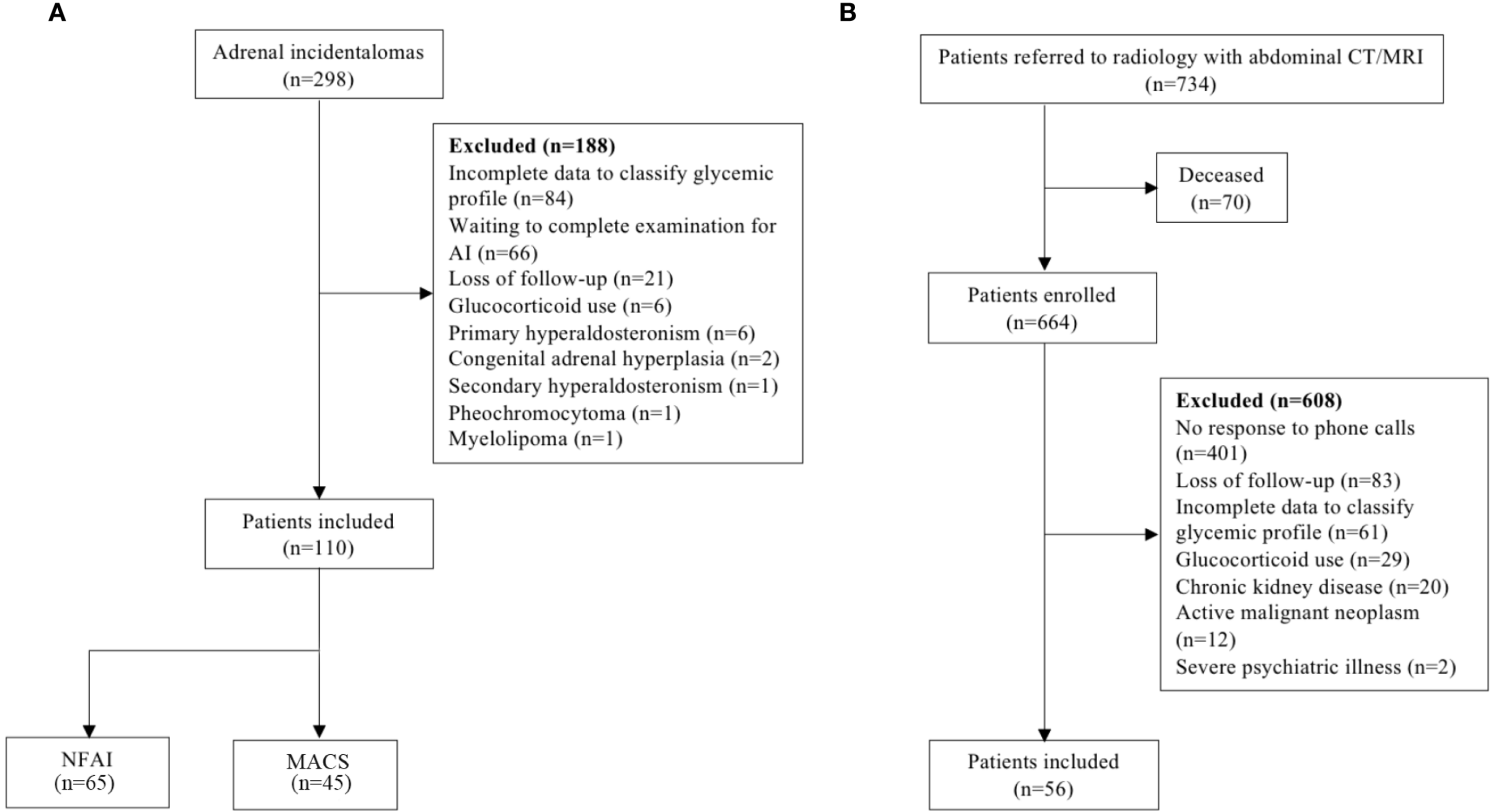
Recruitment strategy flowchart for AI patients **(a)** and control individuals **(b)** participation in the study. *AI*, adrenal incidentaloma; *NFAI*, non-functioning adrenal incidentaloma; *MACS*, mild autonomous cortisol secretion; *CT*, computed tomography; *MRI*, magnetic resonance imaging.

Thus, we selected a convenience sample of all patients with AI followed at the adrenal disease outpatient clinic of HUCFF/UFRJ, after applying the inclusion and exclusion criteria. Sixty-five patients with NFAI, 45 with MACS, and 56 controls were included in the study (**Figure 1**).

### Clinical evaluation

Clinical data and anthropometric measurements were collected for all patients. Ethnicity was classified as white or non-white based on self-reported information. Data on menopausal status (cessation of menstruation for 1 year or more) and smoking status were obtained. Body mass index (BMI) was calculated. Waist circumference (WC) and hip circumference were measured in centimeters, and the waist-to-hip ratio (WHR) was calculated (14).

Glucose metabolism was assessed using FG, OGTT, and glycated hemoglobin (HbA1c). The classification of normoglycemia, prediabetes, and diabetes mellitus (DM) was based on the 2025 American Diabetes Association (ADA) recommendations (15). In addition to the routine assessment of the glycemic profile, other parameters that are still under study were evaluated. The estimated glucose area under the curve (AUC) can be calculated using OGTT data, providing a more complete view of the glycemic response after glucose overload than a single point measurement. Despite its limited use in clinical practice due to low reproducibility, AUC has already been considered a suitable tool for screening for glucose intolerance. The cut-off point of 290 mg h/dL was chosen for the analysis because it has shown high sensitivity (90%) and specificity (93%) to identify cases of IGT and DM in the general population (16). Furthermore, 1h glucose after overload was also analyzed. The cut-off point of 155 mg/dL was chosen in accordance with the International Diabetes Federation (IDF) position statement for the diagnosis of intermediate hyperglycemia, given its important clinical relevance as a predictor of risk for microvascular disease, myocardial infarction, and mortality (17). Individuals with a previous diagnosis of DM did not undergo OGTT. The DM control criteria were defined based on the individualized HbA1c goals for non-pregnant adults, according to ADA recommendations (15). Patients without a prior diagnosis of DM were not using any medication that could interfere with glucose metabolism. Dysglycemia was defined as the presence of pre-DM or DM. Subjects were classified as having dyslipidemia if they were already taking lipid-lowering therapy or had lipid profile levels above the individualized target proposed by the American Association of Clinical Endocrinologists (AACE) (18).

Finally, patients with AI were classified as having or not having MS according to the IDF criteria (19). IDF is the only institution that defines WC based on specific values for each ethnicity. Because there is no specific validation for South Americans, IDF recommends using the same parameters as for South Asians, which was applied in this study (19).

### Laboratory evaluation

Serum cortisol was analyzed using electrochemiluminescence immunoassay (Cobas, Roche 2010, Mannheim, Germany). The inter- and intra-assay coefficients of variation (CV) were 1.6% and 1.3%, respectively.

FG and plasma glucose levels at time 0, 1 hour and 2 hours during OGTT was measured by the enzymatic method using Hexokinase (Roche/Hitachi Cobas c systems) and HbA1C by the HPLC method (Premier Hemoglobin Affinity, Bragança Paulista, Brazil).

The AUC was calculated individually and precisely from the sum of the areas of triangles and rectangles using the trapezoidal method with three points (0, 1h, and 2h) in Microsoft Excel. The unit of AUC is mg·h/dL.

### Statistical analysis

The statistical analyses were performed using SPSS version 23.0 for MacOS (SPSS Inc., Chicago, IL, USA). In the descriptive analysis, categorical variables were expressed as frequencies and percentages, while numerical variables were expressed as means ± standard deviations (SD) or medians (minimum – maximum). Student’s T-test or the Mann-Whitney U-test was used to compare numerical variables between the two groups, as appropriate. The Kruskal–Wallis’s test or ANOVA was used to compare numerical variables among three groups (NFAI, MACS, and controls). In these cases, Bonferroni or Tukey *post-hoc* tests were used to identify significant differences between pairs of groups, accordingly. The chi-square test or Fisher’s exact test was applied to compare categorical variables. Correlations between numerical variables were analyzed using the Spearman test. Binary logistic regression was used to explore the influence of each independent variable (AI, age, BMI, and WC] on AUC > 290 mg h/dL and (AI, age, BMI, and WHR] on the relevance of OGTT to correctly classify glycemic alterations. A p-value < 0.05 was considered statistically significant.

## Results

### Demography, clinical, and laboratorial characteristics in patients with NFAI, MACS, and controls

There were no significant differences among the three groups regarding age, gender, ethnicity, and menopause. Patients with NFAI had a higher BMI and WC than controls. The BMI and WC were similar between NFAI and MACS groups. Patient’s clinical and anthropometric characteristics, and laboratory data are described in **Table 1**.

**Table 1:** Clinical, anthropometric, and laboratory data of the study groups.

Control group	Adrenal incidentaloma		Controls (n = 56)	*p* value*
	NFAI (n = 65)	MACS (n =45)		
Age (years)	57.3 ± 10.0	60.9 ± 10.3	60.3 ± 12.9	0.2
Gender (% women)	80.0	84.4	71.4	0.3
Race (% White)	52.5	61.4	65.5	0.2
Smoking (%)	24.2	20.5	12.7	0.3
Menopause (%)	80.0	88.9	80.0	0.5
Hypertension (%)	78.1	86.7‡	66.1‡	**0.04**
Dyslipidaemia (%)	89.2 †	80.0	73.2 †	0.07
Previous T2DM (%)	47.7	42.2	42.9	0.4
BMI (kg/m^2^)	32.0 ± 5.2 †	31.0 ± 5.8	29.0± 6.5 †	**0.02**
WC (cm)	104.4 ± 10.8 †	100.4 ± 12.2	97.3± 15.3 †	**0.01**
WHR	0.96 ± 0.1 †	0.93 ± 0.1	0.93 ± 0.1 †	**0.02**
Dysglycemia (%)	90.8 †	91.1 ‡	73.2 †‡	**0.01**
Fasting Glucose (mg/dL)	108.0 (71.0 – 327.0)	106.0 (82.5 – 315.5)	104.0 (74.5 – 185.0)	0.2
HbA1c (%)	6.1 (5.0 – 11.1)	6.1 (5.0 – 12.3)	6.1 (4.9 – 9.0)	0.8
AUC OGTT 75g (mg h/dL)	287.6 ± 56.6 †	310.6 ± 58.8 ‡	239.5 ± 58.7 †‡	**0.002**
AUC > 290 mg h/dL (%)	55.0 †	75.0 ‡	22.2 †‡	**0.008**
Glucose ≥ 155 mg/dL 1h OGTT 75g (%)	65 †	75 ‡	27.8 †‡	**0.012**
OGTT 75 changed dysglycemia classification (%)	23 †	27 ‡	3 †‡	**0.03**
MS (IDF criteria) (%)	90.5 †	91.1 ‡	64.3 †‡	**< 0.001**
DHEAS (ng/dL)	65.4 (10.0 – 188.0)	47.0 (5.0 – 2076.0)	**-**	0.7
1 mg DST (mmol/L)	1.1 (0.6 – 1.8)	2.7 (1.9 – 12.7)	**-**	**<0.001**
ACTH (pmol/L)	17.4 ± 11.2	13.6 ± 8.8	**-**	0.1
Tumor Size (cm)	1.9 ± 0.8	2.7 ± 1.0	**-**	**<0.001**

Normally distributed data were presented using the mean (± SD) and non-normally distributed data were presented using the median (minimum and maximum).

*p*-value*: Kruskal–Wallis test or ANOVA among the three groups (controls, NFAI and MACS); Bonferroni or Tukey *post-hoc* tests were used to identify which of the pairs of groups differ from each other, accordingly. †Controls *vs*. NFAI; ‡ Controls *vs.* MACS. There were no differences between NFAI and ACS regarding the variable studied.

*NFAI*, non-functioning adrenal incidentaloma; *MACS, mild* autonomous cortisol secretion; *T2DM*, type 2 diabetes mellitus*; BMI*, body mass index; *WC*, waist circumference; *WHR*, waist-to-hip ratio; *HbA1C g*,lycated hemoglobin*; AUC OGTT 75g*, estimated glucose area under the curve calculated using oral glucose tolerance test with 75g of dextrose data; *OGTT 75g*, oral glucose tolerance test with 75g of dextrose; *MS*, metabolic syndrome; *DHEAS*, dehydroepiandrosterone sulphate; *1-mg DST*, suppression test with 1 mg dexamethasone; *ACTH, adrenocorticotrophic hormone*.The number in bold corresponds to values with statistical significance.

**Figure 2:**
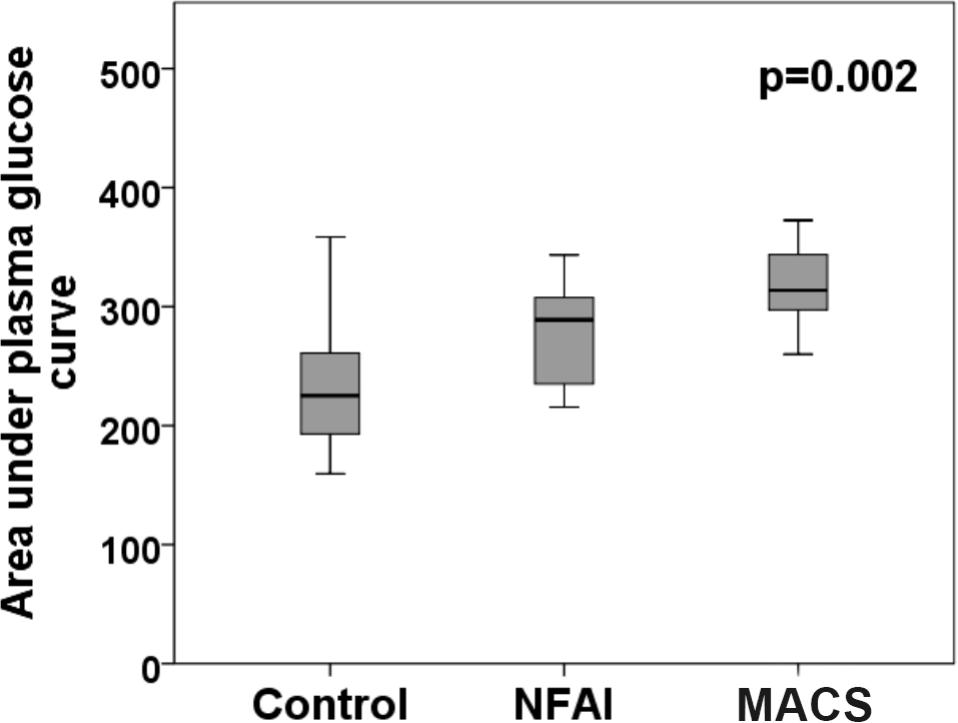
Area under the curve was higher in the MACS and NFAI groups when compared to control group (310.6 *vs*. 287.6 *vs*. 239.5 mg h/dL, respectively; p=0.002). *NFAI*, non-functioning adrenal incidentaloma; *MACS*, mild autonomous cortisol secretion.

AUC was higher in the MACS and NFAI groups compared to the control group (310.6 *vs*. 287.6 *vs*. 239.5 mg h/dL, respectively; p=0.002) (**Figure 2**). An AUC greater than 290 mg h/dL was observed in 75% of patients in the MACS group and in 55% in the NFAI group, without a statistically significant difference between groups. In the control group, the percentage of patients with an AUC greater than 290 mg h/dL was 22%, lower than both groups (p**=**0.008). Plasma glucose level ≥155 mg/dL at 1 hour during the OTGG were observed in 75% of patients with MACS, 65% of NFAI, and 28% of controls (p=0.01).

The frequency of dysglycemia (pre-DM and DM) was higher in the MACS and NFAI groups compared to the control group (91.1 *vs*. 90.8 *vs*. 73.2%, respectively; p=0.01). The number of patients with a prior diagnosis of DM was 24 (42.9%) in the control group, 31 (47.7%) in the NFAI group, and 19 (42.2%) in the MACS group, a finding that was not statistically significant between the groups. The distribution of normoglycemia, pre-DM, and DM was as follows: controls: 26,8%, 25%, and 48,2%; NFAI: 9,2%, 40%, and 50,8%; MACS: 8,9%, 40%, and 51,1%, respectively.

The OGTT was performed in 26 individuals in the MACS group, 34 in the NFAI group and 32 in the control group. It was able to change the dysglycemia classification in 27% of patients with MACS and 23% with NFAI, while in the control group, it changed only in 3% (p=0.03).

**Figure 3:**
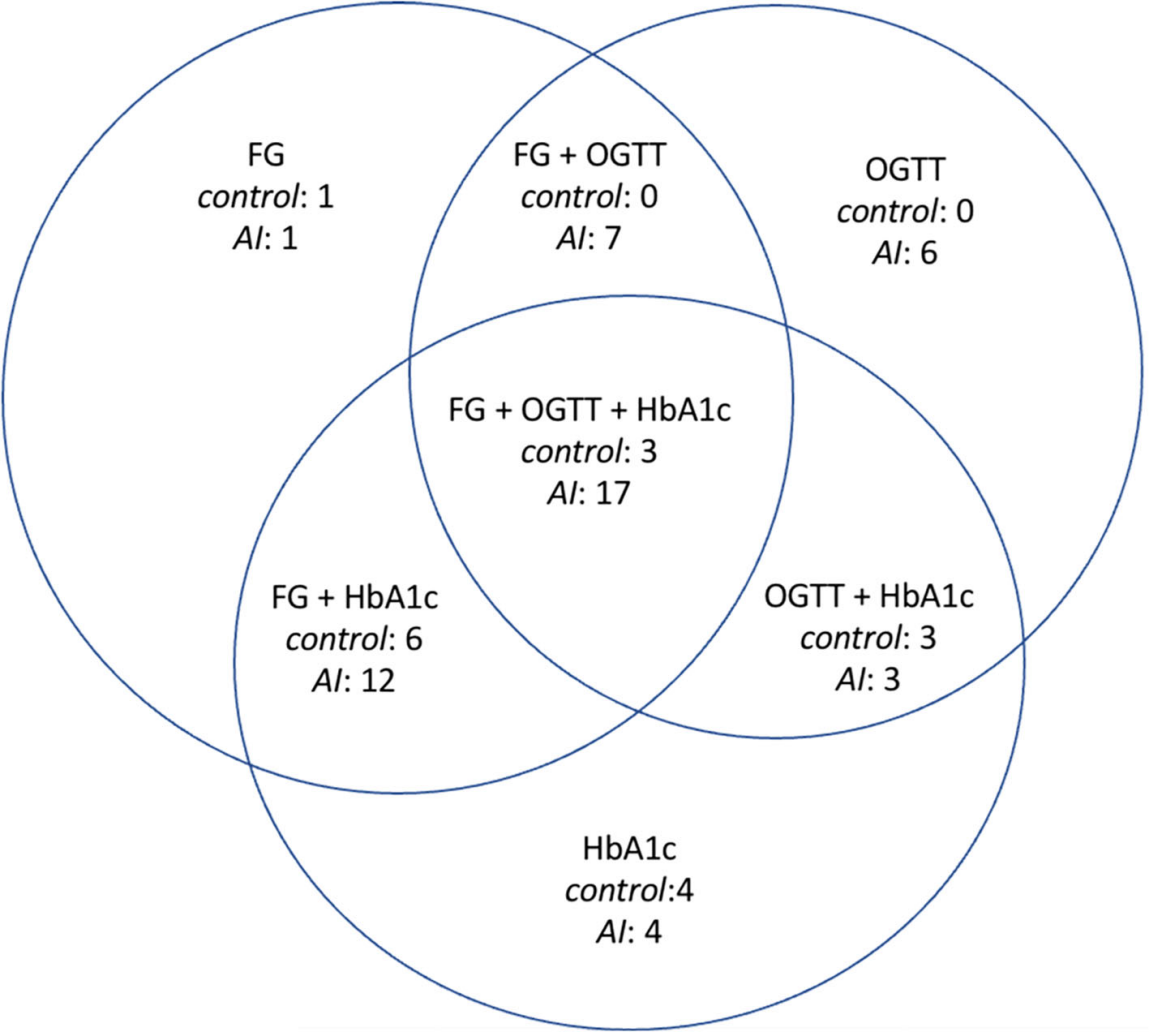
Glycemic alterations found in those individuals who do not have a previous diagnosis of diabetes. FG, only altered fasting glucose; OGTT, only impaired glucose tolerance; HbA1c, only altered glycated hemoglobin; FG+OGTT, fasting glucose altered and impaired glucose tolerance; FG+HbA1c, altered fasting glucose and altered glycated hemoglobin; OGTT+HbA1c, impaired glucose tolerance and altered glycated hemoglobin; FG+OGTT+HbA1c, altered fasting glucose, impaired glucose tolerance, and altered glycated hemoglobin, AI, adrenal incidentaloma.

The **Figure 3** shows in detail the glycemic alterations found in individuals without a previous diagnosis of DM, comparing controls and AI patients.

DM was uncontrolled in half of the patients with AI, while in the control group, 63.6% of individuals had satisfactory control, although this difference was not statistically significant.

**Figure 4:**
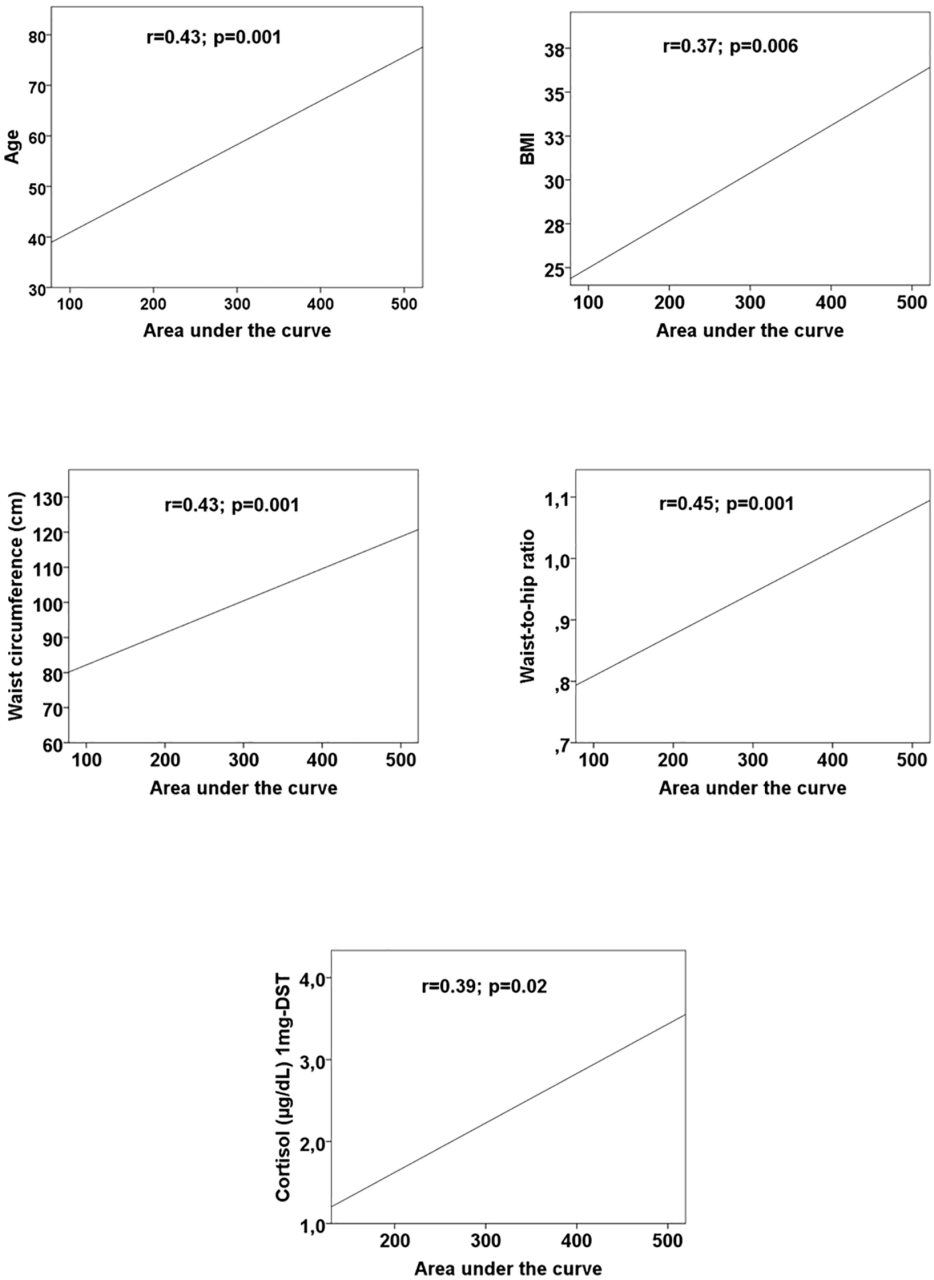
Scatter plots with regression lines between area under the curve and age, body mass index (BMI), waist circumference, waist-to-hip ratio, and cortisol after 1mg-dexamethsasone suppression test. *BMI*, body mass index; *1-mg DST* suppression test with 1 mg dexamethasone.

We found positive correlations between AUC and: cortisol levels after 1mg-DST (r=0.39; p=0.02), age (r=0.43; p=0.001), BMI (r=0.37; p=0.006), WC (r=0.43; p=0.001), and WHR (r=0.45; p=0.001) (**Figure 4**).

The ROC curve analysis showed that the cortisol level on 1mg-DST was not a good predictor of presence of dysglycemia in patients with IA (AUC < 0.7).

### Multivariate analysis

Binary logistic regression analysis was performed to identify independent variables associated with the AUC greater than 290 mg h/dL. The model showed that the presence of AI was a determinant factor, whereas age, BMI and WC were not. The presence of AI increased the odds ratio for a subject to have an AUC > 290 mg h/dL by 5.9 times (**Table 2**).

**Table 2:** Binary logistic regression analysis to predict AUC greater than 290 mg h/dL.

Variables	OR	β	*P-value*
AI	5.92	1.78	**0.03**
Age	1.05	0.05	0.08
BMI	0.94	0.06	0.66
WC	1.05	0.05	0.31

*p-value*: significance level < 0.05.

*AUC*, area under the curve*; AI*, adrenal incidentaloma; *BMI*, body mass index; *WC*, waist circumference; *OR*, odds ratio; *B*, regression coefficient.The number in bold corresponds to values ​​with statistical significance.

Binary logistic regression analysis was also performed to identify independent variables associated with the relevance of OGTT to correctly classify glycemic alterations. The model showed that the presence of AI increased the odds ratio for a subject to be benefit from OGTT for more accurate dysglycemia classification by 9.5 times (**Table 3**).

**Table 3:** Binary logistic regression analysis to predict the relevance of OGTT to correctly classify glycemic alterations.

Variables	OR	β	*P-value*
AI	9.51	2.25	**0.03**
Age	1.01	0.06	0.83
BMI	1.04	0.03	0.57
WHR	12.27	2.66	0.55

*p-value*: significance level < 0.05.

*OGTT*, oral glucose tolerance test*; AI*, adrenal incidentaloma; *BMI*, body mass index; *WHR*, waist hip ratio; *OR*, odds ratio; *B*, regression coeficient.The number in bold corresponds to values with statistical significance.

## Discussion

Patients with MACS have a higher incidence of central obesity, IGT, MS, and DM (5–7). Recent studies have also shown an increase in these conditions in patients with NFAI (8). Robust data indicate that patients with overt hypercortisolism develop glycemic changes primarily through mechanisms that induce glucose intolerance; therefore, OGTT is considered crucial in this population (10, 11). Our results showed that dysglycemia frequency was higher in MACS and NFAI than controls and the use of OGTT was determinant for a correct classification of glycemic status.

Although only the 2-hour glucose measurement in OGTT is considered a diagnostic criterion for IGT and DM according to the 2025 ADA recommendations, other parameters in OGTT seem to be highly relevant (15, 20). The glucose AUC is an index of the overall glucose excursion during the test and may provide more information than a single plasma glucose measurement (16). Here, we clearly demonstrated that the glucose AUC during OGTT was higher in the MACS and NFAI groups compared to controls. A previous study in patients with AI and serum cortisol level < 138 nmol/L (< 5.0 μg/dL), using the 1mg-DST, demonstrated an increased AUC only in patients older than 45 years and with a BMI greater than 30 kg/m^2^ compared to age- and BMI-matched controls (21).

In the general population, glucose AUC values in OGTT greater than 290 mg h/dL have high sensitivity and specificity for detecting cases of IGT and DM (16). Interestingly, in our study, the presence of AI was a determinant factor for AUC values >290 mg h/dL, regardless of age, BMI, or WC, with 75% of patients with MACS, 55% of NFAI, and only in 22% of controls reaching these values.

Another OGTT parameter that has been extensively studied in the general population is the 1-hour plasma glucose measurement, which was recently incorporated by the IDF as a diagnostic criterion for intermediate hyperglycemia and DM (17). Glucose values at this time point have already been identified as a good predictor of incident DM, and plasma glucose values greater than 155 mg/dL at 1-hour during OGTT have been associated with an adverse cardiovascular risk profile, including higher BMI, abdominal obesity, carotid atherosclerosis, and fatty liver (22). The IDF now proposes that values at or above this cutoff point are diagnostic for intermediate hyperglycemia (17). Terzolo et al. found higher plasma glucose values at 1-hour during OGTT in patients with AI compared to controls (23). In our results, both the MACS and NFAI groups had a higher percentage of patients with plasma glucose levels greater than or equal to 155 mg/dL at the 1 hour during OGTT compared to controls. To the best of our knowledge, this is the first study to evaluate the 155 mg/dL cutoff for 1-hour plasma glucose in a population of patients with MACS and NFAI.

Cardiovascular disease is the leading cause of death worldwide, so the ability to identify patients at higher risk of future cardiovascular events is crucial (24). Several studies have found a higher frequency of cardiovascular risk factors in both MACS and NFAI (9, 25). Morelli et al. also showed a higher prevalence of cardiovascular events in MACS, and Di Dalmazi et al. even demonstrated increased cardiovascular mortality in these patients (6, 26). Although many questions remain about the prevalence of cardiovascular disease and mortality in NFAI, it is increasingly clear that these are not innocuous conditions (6, 7). Calavari et al. have already described a higher frequency of cardiovascular risk factors, such as pre-DM, dyslipidaemia, hypertension, and higher WC and MS frequency, in NFAI (8).

As described above, our study demonstrated a higher frequency of another parameter associated with a worse cardiovascular profile in patients with AI compared to the control group. Furthermore, we showed that the frequency of patients with plasma glucose levels greater than or equal to 155 mg/dL at the 1-hour during the OGTT was similar between the MACS and NFAI groups, suggesting that NFAI may be as harmful as MACS in terms of cardiovascular disease. This finding aligns with the results of the study by Rebelo et al., which showed that both NFAI and MACS presented higher frequencies of cardiometabolic morbidities compared to controls, with no significant difference between them (27). To further elucidate this hypothesis, longitudinal studies are needed.

Additionally, the frequency of dysglycemia was similar between in MACS and NFAI groups but significantly higher than controls. In the literature, some studies have shown a higher frequency of DM in patients with MACS compared to patients with NFAI, such as the studies by Di Dalmazi et al. and Morelli et al. (6, 26). However, the study by Terzolo et al. found no difference in the frequency of dyglycemia between MACS and NFAI, similar to our findings (28). Several studies have shown a higher frequency of DM in patients with NFAI compared to the general population. In the Athanasouli et al. meta-analysis, patients with NFAI had a twofold higher risk of having DM than controls (29). These findings suggest that glucose alterations should be monitored not only in patients with MACS but also in patients with NFAI.

The frequency of dysglycemia found in our study (greater than 90%) is higher than that reported in previous studies (9, 30). We hypothesize that this may be due to two reasons: 1) patient population - our hospital is a quaternary care hospital, serving patients with high morbidity. As a result, the frequency of dysglycemia found in the control group was also higher than that observed in the general population with similar age and living in geographically close urban areas of Rio de Janeiro with similar socioeconomic and environmental factors. 2) Diagnostic criteria - our study used all three tests recommended by the ADA for pre-DM and DM screening, which may have increased the diagnostic accuracy. Indeed, a study by Krzyzewska et al., which also used the three tests, showed normoglycemia in less than 30% of patients with NFAI (31).

As DM is a major cardiovascular risk factor, its accurate detection is critical for early diagnosis and treatment to prevent cardiovascular disease. Additionally, the presence of DM was a relevant predictor of hypothalamic-pituitary-adrenal axis recovery in patients with non-aldosterone-producing adrenocortical adenoma undergoing surgery, highlighting the importance of appropriate screening in this population (15, 32).

The OGTT was crucial for correctly classifying glycemic alterations in approximately 25% of AI patients, whereas in the control group, we observed a benefit in only 3%. In other words, patients with AI were 9.5 times more likely to have their dysglycemia classification changed by OGTT compared to the general population, regardless of age, BMI, and WHR. It is also important to emphasize that OGTT was decisive even for patients with normal FG and HbA1c. The OGTT is recognized as a more sensitive test for detecting glycemic alterations than FG and HbA1c alone, enabling the detection of pre-DM and DM in individuals who would otherwise be misclassified as normoglycemic (15). It is known that OGTT identifies a largest number of previously undiagnosed DM patients with established coronary artery disease (33). Our study demonstrated the importance of OGTT in classifying dysglycemia in a population of patients with AI, changing the classification in 27% of patients with MACS and 23% of patients with NFAI, compared to only 3% of the control group. This reinforces the clinical utility of OGTT to avoid underdiagnosis. It is important to emphasize that studies demonstrating the importance of OGTT in patients with AI are still lacking (10, 11). To the best of our knowledge, this is the first study that objectively demonstrates the importance of OGTT in the dysglycemia classification in a population of patients with AI.

Very recently, the IDF proposed shortening the OGTT from 2 to 1 hour, claiming that this would not reduce the informativeness of the test but would offer advantages (17). The suggested diagnostic values for plasma glucose levels at 1-hour greater than or equal to 155 mg/dL for pre-DM and greater than or equal to 209 mg/dL for DM are already being used by the Brazilian Diabetes Society, as these cutoff points result in earlier detection of DM and pre-DM (34). In fact, using these criteria in our study population, 16.7% of patients with AI would change their glycemic status classification, while 11.1% of individuals in the control group would have their classification changed (p=0.7).

A cortisol level cutoff on the 1mg-DST as a predictor of dysglycemia could not be defined using ROC curve analysis, as the area under the curve was not appropriate. This finding may be due to a small sample size or the fact that a cortisol level cutoff to dichotomize this variable may not be adequate to predict dysglycemia. Thus, in light of these results and several published studies, cortisol levels on the 1mg-DST should be interpreted as a continuous rather than a categorical variable (1, 35). In fact, both patients with MACS and NFAI exhibited similar glycemic alterations in the current study. Furthermore, it is hypothesized that patients with NFAI may have a minimally increased secretion of intermediates of the gluco- and/or mineralocorticoid pathway, undetected by current clinical methods, but sufficient to cause metabolic alterations (1, 35). Another hypothesis is an alteration in the circadian rhythm of cortisol secretion, which may contribute to the worsening of metabolic disorders, without evident laboratory alterations (36). Once again, this finding supports the notion that NFAI are not harmless lesions, as previously thought (6, 7). The classification of adrenal incidentalomas as “non-functional” may indeed be an inadequate and misleading term, as it minimizes this potential continuum of hormone secretion which translates clinically in a continuum of glucose impairment (37). Although these tumors do not meet the diagnostic criteria for overt hypercortisolism or Cushing’s syndrome, recent studies suggest that they may secrete inappropriate amounts of glucocorticoid that are not detected by our current clinical practice and nevertheless contribute to adverse outcomes (38). Despite presenting fewer glycemic alterations than Cushing’s syndrome and MACS, in fact, they present more glycemic alterations when compared to a control group (without adrenal tumor) (37, 38).

Our findings, in conjunction with the literature, underscore the importance of recognizing AI as independent risk factors for the development of DM and other glycemic alterations. This suggests the need for more frequent surveillance for glucose intolerance in these patients. In the future, studies that include broad adrenal steroid metabolite profiling will be essential to investigate whether NFAIs secrete hormonal amounts that, while discrete, are sufficient to impact glucose metabolism and contribute to cardiometabolic risk.

Although recent evidence suggests a specific effect of cortisone on each sex, with an earlier onset of glycemic alterations in women, our small sample did not allow us to perform a separate analysis between the sexes with due statistical power (39–41).

Some other limitations of our study should be considered. It is important to emphasize that the control group was selected from a quaternary hospital serving as the state reference for rare, severe, complex and/or advanced-stage diseases. This fact justifies the high frequency of cardiometabolic morbidities in this group, as mentioned earlier. However, the presence of a significantly higher frequency of dysglycemia in patients with AI, even when compared to a control group with a high burden of comorbidities, suggests that our findings are robust. The small sample size of the control group was primarily due to the exclusion criteria. Furthermore, the limited sample size per group may have reduced statistical power, which might explain the absence of differences between MACS and NFAI and the wide confidence intervals observed in the regression analyses. Finally, the lack of cortisol measurements in the control group is also a limitation that should be considered. In fact, we were not authorized by the Institutional Review Board to administer the 1mg-DST to the control group. It is important to note that the AUC was one of the parameters used to assess the glycemic profile, although it has low clinical reproducibility (16). The cross-sectional design limits the evaluation of confounding variables such as lifestyle factors (diet and physical activity), but the use of medications, including antihypertensives and lipid-lowering agents, was systematically documented for all participants and the frequency of these medications was found to be similar in the groups. Ultimately, this study design also limited the ability to draw cause-effect inferences from the observed findings, so longitudinal studies are needed to validate our hypotheses.

In conclusion, our findings demonstrate that patients with AI have a high frequency of dysglycemia, and OGTT significantly enhances the classification of these alterations in a considerable number of patients. Based on our results, we suggest that screening for glycemic alterations be performed in both patients with MACS and NFAI. In this scenario, OGTT should be highlighted as an essential test to ensure an adequate evaluation, even for patients with normal FG and HbA1c. This practice can optimize opportunities for early detection and prevention. Although follow-up studies are needed, it would be possible to infer that a glycemic alteration screening strategy in patients with AI that routinely includes OGTT could, in the future, reduce the rate of DM complications in these patients.

## Data Availability

The raw data supporting the conclusions of this article will be made available by the authors, without undue reservation.

## References

[1] Fassnacht M Tsagarakis S Terzolo M Tabarin A Sahdev A Newell-Price J . European Society of Endocrinology clinical practice guidelines on the management of adrenal incidentalomas, in collaboration with the European Network for the Study of Adrenal Tumors. Eur. J. Endocrinol. (2023) 189:G1–G42. doi: 10.1093/ejendo/lvad066 37318239

[2] Zeiger MA Siegelman SS Hamrahian AH . Medical and surgical evaluation and treatment of adrenal incidentalomas. J. Clin. Endocrinol. Metab. (2011) 96:2004–15. doi: 10.1210/jc.2011-0085 21632813

[3] Zeiger MA Thompson GB Duh QY Hamrahian AH Angelos P Elaraj D . AACE/AAES Medical Guidelines for the management of adrenal incidentalomas. Endocr. Pract. (2009) 15 Suppl 1:1–20. doi: 10.4158/EP.15.S1.1 19632967

[4] Lee JM Kim MK Ko SH Koh JM Kim BY Kim SW . Clinical guidelines for the management of adrenal incidentaloma. Endocrinol. Metab. (Seoul). (2017) 32:200–18. doi: 10.3803/EnM.2017.32.2.200 28685511 PMC5503865

[5] Mantero F Arnaldi G . Management approaches to adrenal incidentalomas. A view from Ancona, Italy. Endocrinol. Metab. Clin. North Am. (2000) 29:107–25. doi: 10.1016/s0889-8529(05)70119-5 10732267

[6] Morelli V Reimondo G Giordano R Casa SD Policola C Palmieri S . Long-term follow-up in adrenal incidentalomas: an Italian multicenter study. J. Clin. Endocrinol. Metab. (2014) 99:827–34. doi: 10.1210/jc.2013-3527 24423350

[7] Taya M Paroder V Bellin E Haramati LB . The relationship between adrenal incidentalomas and mortality risk. Eur. Radiol. (2019) 29:6245–55. doi: 10.1007/s00330-019-06202-y 30993434 PMC6801004

[8] Cavalari EMR Paula MP Arruda M Carraro N Martins A Souza K . Nonfunctioning adrenal incidentaloma: A novel predictive factor for metabolic syndrome. Clin. Endocrinol. (Oxf). (2018) 89:586–95. doi: 10.1111/cen.13822 30044007

[9] Tauchmanova L Rossi R Biondi B Pucrano M Nuzzo V Palmieri EA . Patients with subclinical Cushing's syndrome due to adrenal adenoma have increased cardiovascular risk. J. Clin. Endocr. Metab. (2002) 87:4872–8. doi: 10.1210/jc.2001-011766 12414841

[10] Scaroni C Zilio M Foti M Boscaro M . Glucose metabolism abnormalities in cushing syndrome: from molecular basis to clinical management. Endocr. Rev. (2017) 38:189–219. doi: 10.1210/er.2016-1105 28368467

[11] Barbot M Ceccato F Scaroni C . Diabetes mellitus secondary to cushing’s disease. Front. Endocrinol. (Lausanne). (2018) 9:284. doi: 10.3389/fendo.2018.00284 29915558 PMC5994748

[12] Funder JW Carey RM Fardella C Gomez-Sanchez CE Mantero F Stowasser M . Case detection, diagnosis, and treatment of patients with primary aldosteronism: an endocrine society clinical practice guideline. J. Clin. Endocrinol. Metab. (2008) 93:3266–81. doi: 10.1210/jc.2008-0104 18552288

[13] Lenders JWM Duh QY Eisenhofer G Gimenez-Roqueplo AP Grebe SKG Murad MH . Pheochromocytoma and paraganglioma: an endocrine society clinical practice guideline. J. Clin. Endocrinol. Metab. (2014) 99:1915–42. doi: 10.1210/jc.2014-1498 24893135

[14] Nishida C Ko GT Kumanyika S . Body fat distribution and noncommunicable diseases in populations: overview of the 2008 WHO Expert Consultation on Waist Circumference and Waist–Hip Ratio. Eur. J. Clin. Nutr. (2010) 64:2–5. doi: 10.1038/ejcn.2009.139 19935820

[15] American Diabetes Association Professional Practice Committee . Standards of care in diabetes. Diabetes Care. (2025) 48:S1–S343. doi: 10.2337/dc25-er04b 39651982

[16] Sakaguchi K Takeda K Maeda M Ogawa W Sato T Okada S . Glucose area under the curve during oral glucose tolerance test as an index of glucose intolerance. Diabetol. Int. (2015) 7:53–8. doi: 10.1007/s13340-015-0212-4 30603243 PMC6214468

[17] Bergman M Manco M Satman I Chan J Schmidt MI Sesti G . International Diabetes Federation Position Statement on the 1-hour post-load plasma glucose for the diagnosis of intermediate hyperglycaemia and type 2 diabetes. Diabetes Res. Clin. Pract. (2024) 209:11589. doi: 10.1016/j.diabres.2024.111589 38458916

[18] Jellinger PS Handelsman Y Rosenblit PD Bloomgarden ZT Fonseca VA Garber AJ . American Association of Clinical endocrinologists and American College of Endocrinologist Guidelines for management of dyslipidemia and prevention of cardiovascular disease. Endocr. Pract. (2017) 23:1–87. doi: 10.4158/EP171764.APPGL 28437620

[19] Alberti KG Zimmet P Shaw J . The metabolic syndrome—a new worldwide definition. Lancet. (2005) 366:1059–62. doi: 10.1016/S0140-6736(05)67402-8 16182882

[20] Jagannathan R Neves JS Dorcely B Chung ST Tamara K Rhee M . The oral glucose tolerance test: 100 years later. Diabetes Metab. Syndr. Obes. (2020) 13:3787–805. doi: 10.2147/DMSO.S246062 33116727 PMC7585270

[21] Vierhapper H Heize G Gessl A Exner M . Adrenocortical tumors: prevalence of impaired glucose tolerance and of "Paradoxical Rise" of cortisol during an oral glucose tolerance test. Exp. Clin. Endocrinol. Diabetes. (2003) 111:415–20. doi: 10.1055/s-2003-44288 14614648

[22] Ahuja V Aronen P Pramodkumar TA Looker H Chetrit A Bloigu AH . Accuracy of 1-hour plasma glucose during the oral glucose tolerance test in diagnosis of type 2 diabetes in adults: A meta-analysis. Diabetes Care. (2021) 44:1062–9. doi: 10.2337/dc20-1688 33741697 PMC8578930

[23] Terzolo M Pia A Alì A Osella G Reimondo G Bovio S . Adrenal Incidentaloma: A new cause of metabolic Syndrome? J. Clin. Endocr. Metab. (2002) 87:998–1003. doi: 10.1210/jcem.87.3.8277 11889151

[24] Williams B Mancia G Spiering W Rosei EA Azizi M Burnier M . ESC/ESH guidelines for the management of arterial hypertension: the task force for the management of arterial hypertension of the european society of cardiology and the european society of hypertension: the task force for the management of arterial hypertension of the european society of cardiology and the european society of hypertension. J. Hypertens. (2018) 36:1953–2041. doi: 10.1097/HJH.0000000000001940 30234752

[25] Yener S Ertilav S Secil M Akinci B Demir T Kebapcilar L . Increased risk of unfavorable metabolic outcome during short-term follow-up in subjects with nonfunctioning adrenal adenomas. Med. Princ Pract. (2012) 21:429–34. doi: 10.1159/000336589 22398948

[26] Di Dalmazi G Vicennati V Garelli S Casadio E Rinaldi E Giampalma E . Cardiovascular events and mortality in patients with adrenal incidentalomas that are either non-secreting or associated with intermediate phenotype or subclinical Cushing's syndrome: a 15-year retrospective study. Lancet Diabetes Endocrinol. (2014) 2:396–405. doi: 10.1016/S2213-8587(13)70211-0 24795253

[27] Rebelo JFD Costa JM Junqueira FD Fonseca AO Almeida ABABS Moraes AB . Adrenal incidentaloma: do patients with apparently nonfunctioning mass or autonomous cortisol secretion have similar or different clinical and metabolic features? Clin. Endocrinol. (Oxf). (2023) 98:662–9. doi: 10.1111/cen.14861 36514987

[28] Terzolo M Bovio S Pia A Conton P Reimondo G Dall’Asta C . Midnight serum cortisol as a marker of increased cardiovascular risk in patients with a clinically inapparent adrenal adenoma. Eur. J. Endocrinol. (2005) 153:307–15. doi: 10.1530/eje.1.01959 16061838

[29] Athanasouli F Georgiopoulos G Asonitis N Petychaki F Savelli A Panou E . Nonfunctional adrenal adenomas and impaired glucose metabolism: a systematic review and meta-analysis. Endocrine. (2021) 74:50–60. doi: 10.1007/s12020-021-02741-x 33963515

[30] Di Dalmazi G Vicennati V Rinaldi E Morselli-Labate AM Giampalma E Mosconi C . Progressively increased patterns of subclinical cortisol hypersecretion in adrenal incidentalomas differently predict major metabolic and cardiovascular outcomes: a large cross-sectional study. Eur. J. Endocrinol. (2012) 166:669–77. doi: 10.1530/EJE-11-1039 22267278

[31] Krzyzewska K Niemczuk E Mysliwiec BJ Junik R . Glucose metabolism disorders in patients with non-functioning adrenal adenomas — single-centre experience. Endokrynol Pol. (2017) 68:416–21. doi: 10.5603/EP.a2017.0034 28585681

[32] Bonaventura I Tomaselli A Angelini F Ferrari D De Alcubierre D Hasenmajer V . Predicting postoperative hypocortisolism in patients with non-aldosterone-producing adrenocortical adenoma: a retrospective single-centre study. J. Endocrinol. Invest. (2024) 47:1751–62. doi: 10.1007/s40618-023-02283-1 38386266 PMC11196308

[33] Mann BK Bhandohal JS Hong J . An overall glance of evidence supportive of one-hour and two-hour postload plasma glucose levels as predictors of long-term cardiovascular events. Int. J. Endocrinol. (2019), 6048954. doi: 10.1155/2019/6048954 31929794 PMC6935819

[34] Rodacki M Zajdenverg L Silva Júnior WS Giacaglia L Negrato CA Cobas RA . Brazilian guideline for screening and diagnosis of type 2 diabetes: a position statement from the Brazilian Diabetes Society. Diabetol. Metab. Syndrome. (2025) 17:78. doi: 10.1186/s13098-024-01572-w 40038723 PMC11881304

[35] Tabarin A . Do the diagnostic criteria for subclinical hypercortisolism exist? Annales d’ Endocrinologie. Ann. Endocrinol. (Paris). (2018) 79:146–8. doi: 10.1016/j.ando.2018.03.013 29661471

[36] Di Dalmazi G . Adrenal incidentaloma: picking out the high-risk patients. Exp. Clin. Endocrinol. Diabetes. (2019) 127:178–84. doi: 10.1055/a-0713-0598 30372764

[37] Han MM Cao XM Liu ZA Zhang Y Liu YF . Continuum of glucose and bone metabolism impairment across autonomous cortisol secretion: A cross-sectional study. World J. Diabetes. (2025) 16:100580. doi: 10.4239/wjd.v16.i3.100580 40093276 PMC11885967

[38] Lopez D Luque-Fernandez MA Steele A Adler GK Turchin A Vaidya A . Nonfunctional" Adrenal tumors and the risk for incident diabetes and cardiovascular outcomes: A cohort study. Ann. Intern. Med. (2016) 165:533–42. doi: 10.7326/M16-0547 27479926 PMC5453639

[39] Puglisi S Barač Nekić A Morelli V Alessi Y Fosci M Pani A . Are comorbidities of patients with adrenal incidentaloma tied to sex? Front. Endocrinol. (Lausanne). (2024) 15:1385808. doi: 10.3389/fendo.2024.1385808 38808113 PMC11130385

[40] Bonaventura I Minnetti M Ferrari D Hasenmajer V Tomaselli A De Alcubierre D . Impact of female sex and mild cortisol secretion on coagulation profile in adrenal incidentalomas. J. Endocr. Soc. (2024) 9:bvae215. doi: 10.1210/jendso/bvae215 39669652 PMC11635453

[41] Deutschbein T Reimondo G Di Dalmazi G Bancos I Patrova J Vassiliadi DA . Age-dependent and sex-dependent disparity in mortality in patients with adrenal incidentalomas and autonomous cortisol secretion: an international, retrospective, cohort study. Lancet Diabetes Endocrinol. (2022) 10:499–508. doi: 10.1016/S2213-8587(22)00100-0 35533704 PMC9679334

